# Hierarchical Control of *Drosophila* Sleep, Courtship, and Feeding Behaviors by Male-Specific P1 Neurons

**DOI:** 10.1007/s12264-018-0281-z

**Published:** 2018-09-04

**Authors:** Wenxuan Zhang, Chao Guo, Dandan Chen, Qionglin Peng, Yufeng Pan

**Affiliations:** 1 The Key Laboratory of Developmental Genes and Human Disease, Institute of Life Sciences, Southeast University, Nanjing, 210096 China; 2 Co-innovation Center of Neuroregeneration, Nantong University, Nantong, 226019 China

**Keywords:** *Drosophila*, Courtship, Sleep, Feeding, P1 neurons, Neural circuit

## Abstract

Animals choose among sleep, courtship, and feeding behaviors based on the integration of both external sensory cues and internal states; such choices are essential for survival and reproduction. These competing behaviors are closely related and controlled by distinct neural circuits, but whether they are also regulated by shared neural nodes is unclear. Here, we investigated how a set of male-specific P1 neurons controls sleep, courtship, and feeding behaviors in *Drosophila* males. We found that mild activation of P1 neurons was sufficient to affect sleep, but not courtship or feeding, while stronger activation of P1 neurons labeled by four out of five independent drivers induced courtship, but only the driver that targeted the largest number of P1 neurons affected feeding. These results reveal a common neural node that affects sleep, courtship, and feeding in a threshold-dependent manner, and provide insights into how competing behaviors can be regulated by a shared neural node.

**Electronic supplementary material:**

The online version of this article (10.1007/s12264-018-0281-z) contains supplementary material, which is available to authorized users.

## Introduction

A fundamental question in biology is how animals sense environmental cues and alter their physiology and behavior in ways that are beneficial for their survival and reproduction. The amenability of *Drosophila melanogaster* as a model system using genetic and physiological approaches makes it ideal for exploring the neural mechanisms underlying a variety of behaviors including sleep, courtship, and feeding. Indeed, substantial progress has been made on how these individual behaviors are controlled by specific neuronal circuits, often referred to as sleep circuits, courtship circuits, and feeding circuits [1–6].

Sleep, courtship, and feeding behaviors are mutually exclusive in principle. Very little is known about how these behaviors cross-talk at the level of neuronal circuits. Recently, we showed that sleep and sexual behaviors interact in a sex-specific way and identified sexually dimorphic neurons that mediate the interplay between sleep and sex [7]. Among these neurons, P1 neurons are of particular interest because they serve as a higher center that integrates both external sensory cues and social experience [8–12]. It has also been found that P1 neurons regulate male aggression, as a P1-activated male fly is more aggressive if presented with another male [13]. P1 neurons are only present in males and express the male-specific Fruitless (FruM) and Doublesex (DsxM) proteins [11, 14–16], which are crucial for male sexual development and behaviors [17–20]. It is generally accepted that the activity of P1 neurons is positively correlated with male sexual arousal in flies [8, 10, 15]. However, it is still unclear whether P1 neurons represent a general arousal state and modulate many other behaviors, and if so, how multiple behaviors are controlled by the same set of neurons.

Here, we used five independent driver lines that labeled P1 neurons ranging from 9 to 23 cells per hemisphere (20%–50% of all P1 neurons) to determine how P1 activation affects sleep, courtship, and feeding behaviors in *Drosophila*.

## Materials and Methods

### Fly Stocks

Flies were maintained at 22°C under a 12 h:12 h light:dark cycle. *Split-GAL4* reagents including *R15A01-AD*, *R17D06-AD*, *R71G01-DBD*, and *R22D03-DBD*, as well as *R17D06-LexA* and *R71G01-LexA* have been described previously [21, 22] and were obtained from Janelia Research Campus (Ashburn, VA). *dsx*^*GAL4*^, *UAS>stop>dTrpA1*, *UAS>stop>myrGFP*, and *LexAop2-FlpL* were used as previously described [15, 23].

### Sleep Test and Analysis

Individual 2–4 day-old male flies were placed in locomotor activity monitor tubes (DAM2, TriKinetics Inc., Waltham, MA) with fly food (2% agarose and 5% sugar), and entrained under 22°C and 12 h:12 h light:dark conditions for at least 2 days before sleep tests. One day of sleep data were first recorded at 22°C as baseline, then the flies were shifted to 25.5°C, 27°C, 28.5°C, or 30°C for two days, and then returned to 22°C for at least one day. Sleep was analyzed as previously described [7]. Changes in total sleep were calculated as the percentage of sleep change on the first day of temperature shift relative to baseline sleep at 22°C.

### Courtship and Locomotion Assay

We used unilateral wing extension in isolated males to compare courtship induced by P1 activation. Males were individually placed in 2 cm-diameter round chambers with food (2% agarose and 5% sugar) at 25.5°C, 27°C, 28.5°C, or 30°C and video was captured for 24 h, starting at 09:00. We then manually scored the percentage of males displaying unilateral wing extension. The average walking velocity during the 24-h recording was further quantified using the ZebraLab software system (ViewPoint Life Sciences, Montreal, Quebec, Canada).

### Feeding

Feeding was assayed using food with blue dye. In brief, flies were starved for 24 h on 1% aqueous agarose at 22°C, then moved to 25.5°C, 27°C, 28.5°C, or 30°C for 30 min for *dTrpA1* activation. Thereafter, they were transferred to 1% FD&C Blue 1 (Sigma-Aldrich, St. Louis, MO) food (2.5% sucrose, 2.5% yeast extract, and 0.5% agar) for 15 min (between 15:00 and 17:00) at the above temperatures for continuous activation while allowing feeding. To quantify the food intake, the absorbance of the ingested blue dye was measured as previously described [24].

### Tissue Dissection, Staining, and Imaging

We dissected the brains of 4–6 day-old male or female flies in Schneider’s insect medium (Thermo Fisher Scientific, Waltham, MA) and fixed them in 4% paraformaldehyde in phosphate-buffered saline (PBS) for 30 min at room temperature. After 4 × 15-min washes in PAT (0.5% Triton X-100 and 0.5% bovine serum albumin in PBS), tissues were blocked in 3% normal goat serum (NGS) for 60 min, then incubated in primary antibodies diluted in 3% NGS for ~24 h at 4°C, washed (4 × 15-min) in PAT, and incubated in secondary antibodies diluted in 3% NGS for ~24 h at 4°C. Tissues were then washed (4 × 15-min) in PAT and mounted in Vectashield (Vector Laboratories, Burlingame, CA) for imaging. The primary antibodies used were rabbit anti-GFP (1:1000; A11122, Invitrogen, Waltham, MA) and mouse anti-Bruchpilot (1:30; nc82, Developmental Studies Hybridoma Bank, Iowa City, IA). The secondary antibodies used were goat anti-mouse IgG conjugated to Alexa 555 (1:500, A28180, Invitrogen) and goat anti-rabbit IgG conjugated to Alexa 488 (1:500, A11008, Invitrogen). Samples were imaged at 20× magnification on a Zeiss 700 confocal microscope, and processed with ImageJ.

### Statistics

Statistical analysis was performed using Prism GraphPad as indicated in the figure legends.

## Results and Discussion

**Fig. 1:**
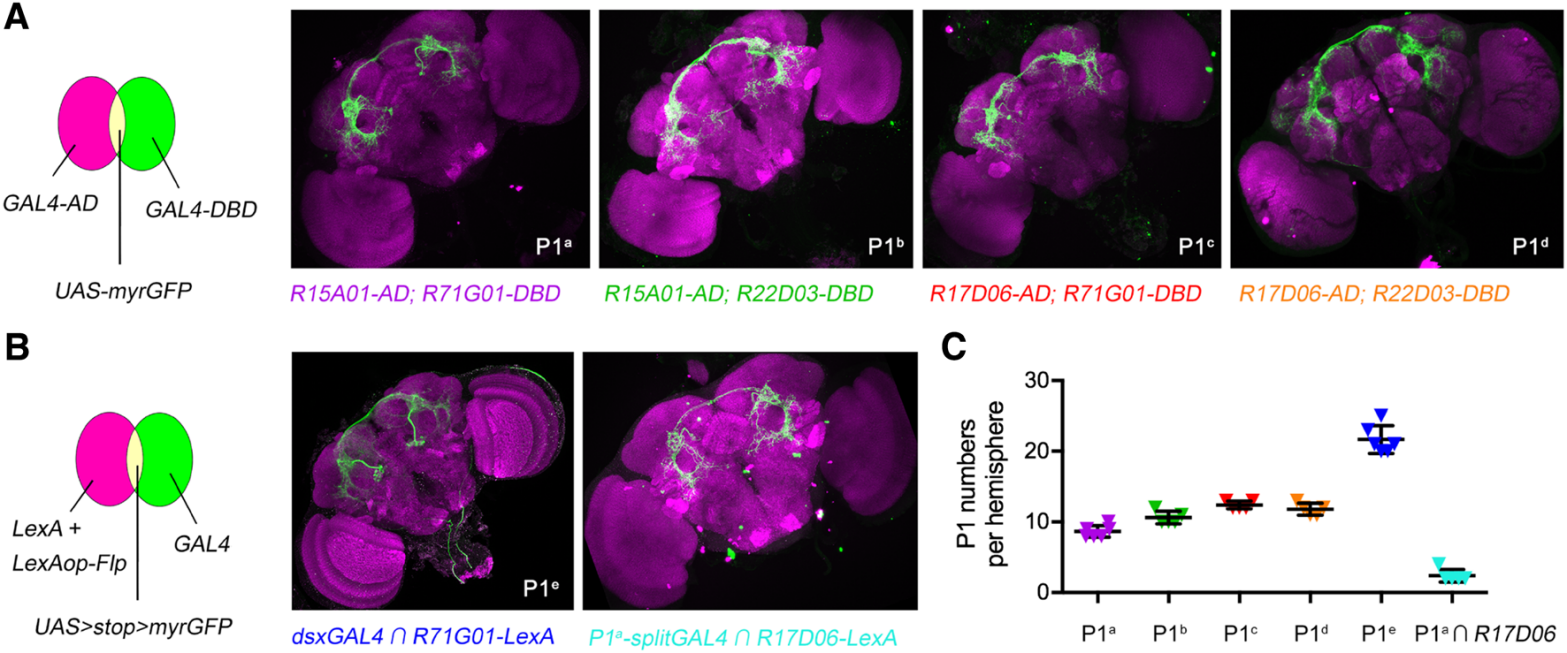
Identification of drivers targeting P1 neurons in male flies. **A** Labeling of P1 neurons in brains of male flies by four *split-GAL4* combinations (P1^a^ by *R15A01-AD; R71G01-DBD*, P1^b^ by *R15A01-AD; R22D03-DBD*, P1^c^ by *R17D06-AD; R71G01-DBD*, and P1^d^ by *R17D06-AD; R22D03-DBD*). **B** Diagram of the FRT/FLP intersectional strategy to label P1^e^ neurons (*R71G01-LexA/LexAop2-FlpL; UAS>stop>myrGFP/dsx*^*GAL4*^). This method also labeled two pairs of P1 neurons with both P1^a^-*splitGAL4* and *R17D06-LexA*. **C** Numbers of P1 neurons labeled in male flies by each of the above driver lines (*n* = 6 for P1^a^ and P1^e^, *n* = 5 for the others; error bars indicate SEM).

To investigate how P1 neurons modulate different behaviors, we first aimed to identify distinct *GAL4* drivers for labeling and manipulating P1 neurons. We previously used two intersectional methods to target P1 neurons, one using the *split-GAL4* system (P1^a^*-splitGAl4*: *R15A01-AD; R71G01-DBD*, Fig. 1A), and the other using the Flip-out system (P1^e^: *R71G01-LexA/LexAop2-FlpL; UAS>stop>myrGFP/dsx*^*GAL4*^, Fig. 1B) [7]. We later identified two fragment *GAL4*s (*R17D06* and *R22D03*) that also label male-specific P1 neurons and thus made three additional *splitGAL4*s (P1^b^ by *R15A01-AD; R22D03-DBD*, P1^c^ by *R17D06-AD; R71G01-DBD*, and P1^d^ by *R17D06-AD; R22D03-DBD*). The four *split-GAL4*s each labeled 9–12 P1 neurons (Fig. 1C), ~20%–25% of all P1 neurons, as well as a few other cells. Although we hoped to target distinct subsets of P1 neurons with different *split-GAL4* drivers, we were not able to discriminate among them. However, when P1^a^-*splitGAl4* that labels 9–10 P1 cells intersected with *R17D06-LexA*, only ~2 P1 neurons were consistently labeled, suggesting that only two P1 neurons are labeled by both drivers. Thus, it was highly likely that these four P1-*splitGAL4* drivers partially overlapped as well as targeting distinct P1 neurons.

**Fig. 2:**
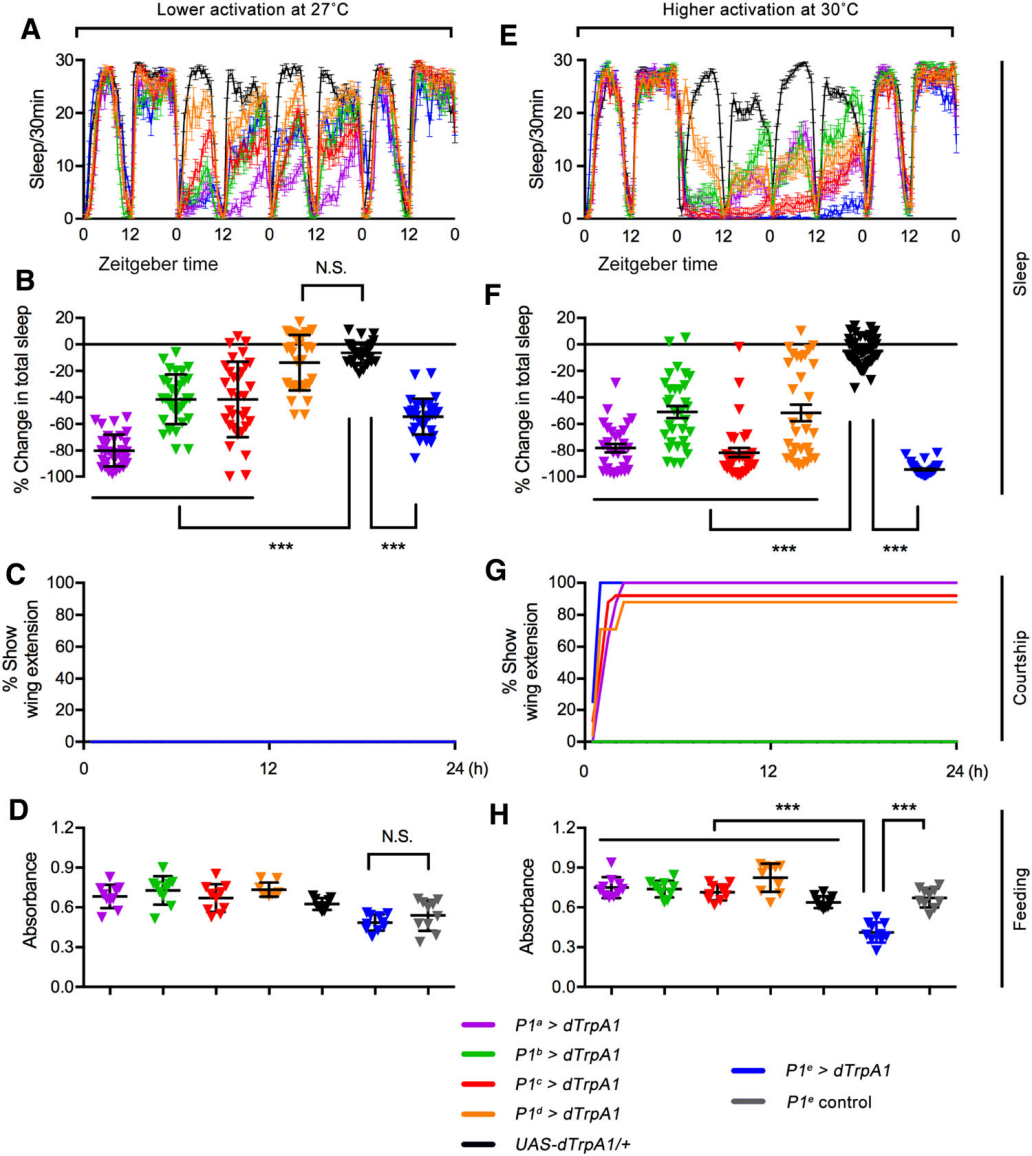
Regulation of sleep, courtship, and feeding behaviors by P1 neurons. **A**–**D** Mild activation of P1 neurons driving dTrpA1 at 27°C using five independent P1 drivers (P1^a^–P1^e^) inhibited sleep (**A**, **B**), but did not affect courtship (**C**) or feeding (**D**) [sleep test at 27°C (**A**, **B**), *n* = 32 each; wing-extension test (**C**), *n* = 48 each; feeding test (**D**), *n* = 10, 10, 10, 9, 9, 10, and 10 (10 flies for each replicate)]. **E**–**H** Stronger activation of P1 neurons at 30°C using all P1 drivers affected sleep (**E**, **F**), while four drivers (P1^a^, P1^c^, P1^d^, and P1^e^) affected courtship (**G**), and only one (P1^e^) affected feeding (**H**) [sleep test at 30°C (**E**, **F**), *n* = 31, 32, 32, 32, 58, and 32; wing-extension test (**G**), *n* = 48 each; feeding test (**H**), *n* = 10 each]. ***P < 0.001, one-way ANOVA. N.S., no significant difference. Error bars indicate SEM.

We next used these five P1 drivers (P1^a^–P1^d^ *splitGAl4*s, and P1^e^ from intersection between *R71G01-LexA* and *dsx*^*GAL4*^, hereafter referred to simply as P1^a^–P1^e^) to investigate how the activation of these neurons *via* the temperature-sensitive activator dTrpA1 [25] modulates sleep, courtship, and/or feeding behaviors. It has been shown that neurons expressing dTrpA1 begin to fire at 26°C, and higher temperatures further increase their activity [26]. Thus we tested the above behaviors at four temperatures (25.5°C, 27°C, 28.5°C, and 30°C), at which P1 neurons would be increasingly activated, presenting data at 27°C as mild activation and 30°C as stronger activation unless stated otherwise. We found that mild activation of P1^a^, P1^b^, P1^c^, and P1^e^, but not P1^d^ neurons significantly inhibited sleep (up to 80% sleep loss, Fig. 2A, B). None of these lines induced unilateral wing extension, a key step in courtship rituals (Fig. 2C), nor did they affect feeding behavior in starved males (Fig. 2D). Furthermore, stronger activation of all P1^a^–P1^e^ neurons at 30°C significantly inhibited sleep (Fig. 2E, F), and such activation of the four sets of P1 neurons (P1^a^, P1^c^, P1^d^, and P1^e^) was able to induce unilateral wing extension (Fig. 2G). However, only stronger activation of the P1^e^ neurons at 30°C significantly suppressed feeding behavior (Fig. 2H). To test whether stronger activation of P1^a^–P1^d^ neurons at a higher temperature affected feeding, we performed the feeding experiments at 32°C, and found the same results as those at 30°C. Activation of P1^e^, but not P1^a^–P1^d^ neurons, suppressed feeding at 32°C (Fig. S1). The P1^e^ driver labeled ~23 P1 neurons, while the other four P1 drivers each labeled 9–12 P1 neurons. There are at least two possibilities why only activation of P1^e^ but not P1^a^–P1^d^ neurons suppressed feeding behaviors in starved males: (1) P1^e^ neurons include a set of neurons that are not labeled by other P1 drivers, and these neurons specifically affect feeding behavior; or (2) as the number of P1 neurons labeled by P1^e^ is about double that of P1^a^–P1^d^, it is possible that feeding requires the activation of a larger number of P1 neurons than sleep or courtship. Together, these results indicate that sleep, courtship, and feeding behaviors are all affected by activation of P1 neurons, but with different activation thresholds.

**Fig. 3:**
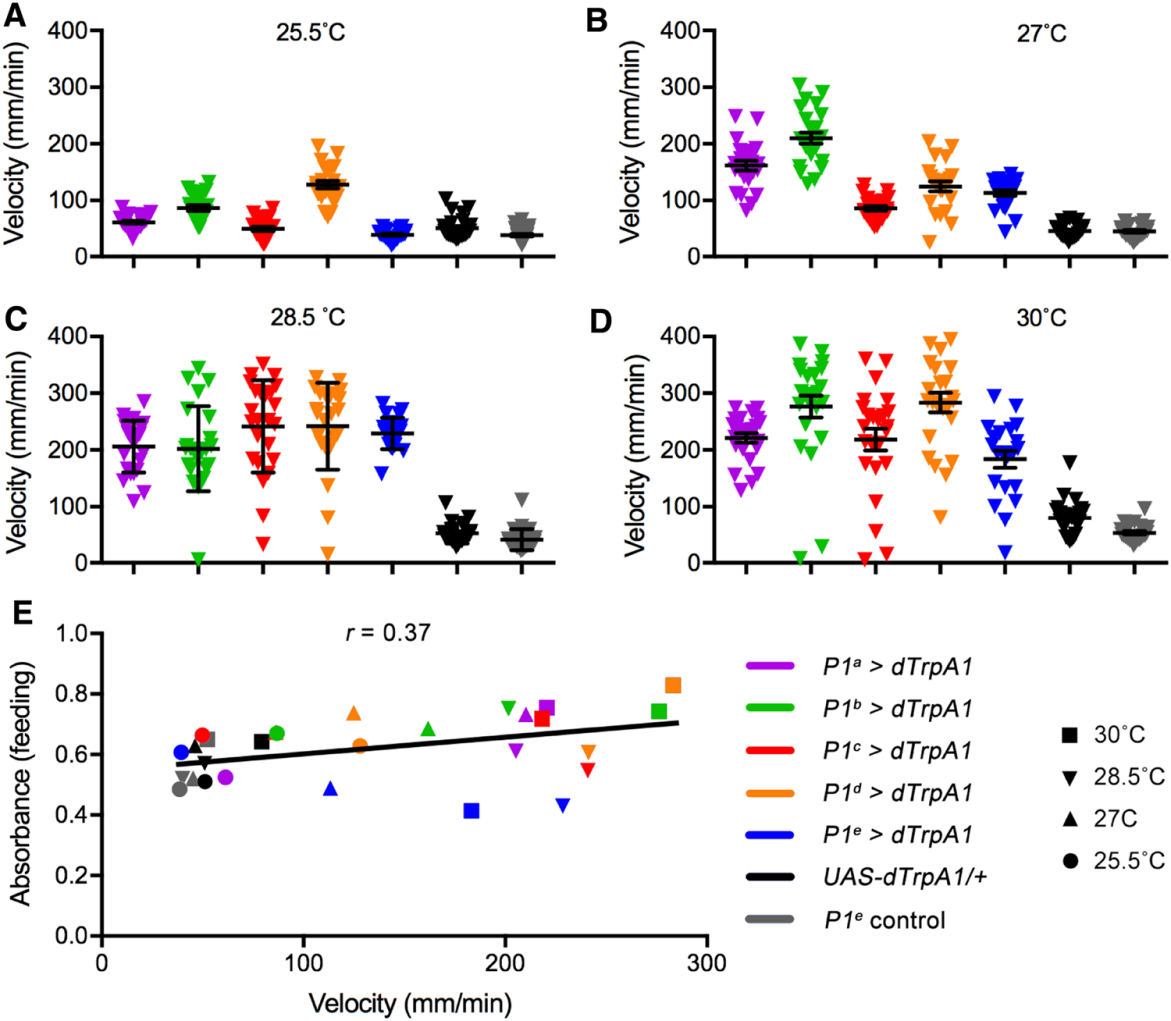
Correlation between feeding behavior and walking velocity in P1-activated male flies. **A**–**D** Mean walking velocity of the indicated genotypes at 25.5°C (**A**), 27°C (**B**), 28.5°C (**C**), and 30°C (**D**) (*n* = 24 each, except that *n* = 21 for P1^e^ activation at 30°C. Error bars indicate SEM). **E** A slightly positive correlation between feeding and walking velocity (*r* = 0.37, *P* = 0.0503, Pearson’s correlation coefficient), so decreased feeding by P1^e^ activation is not due to increased locomotion.

We next asked whether the suppression of feeding by activation of P1^e^ neurons was correlated with locomotor activity, as stronger activation of P1^e^ neurons at 30°C indeed increases walking velocity [7]. We assayed walking velocity in all P1^a^–P1^e^-activated males at various temperatures (25.5–30°C, Fig. 3A–D) for 24 h, and found that mild activation at 27°C already increased the average velocity in P1^e^-activated males, as well as in the other P1-activated males. Stronger activation at 30°C further increased the velocity, but that of P1^e^-activated males was not as high as the other P1-activated males (average velocity of P1^a^–P1^e^-activated males: 221.0 ± 8.4, 266.6 ± 19.1, 218.6 ± 19.3, 283.5 ± 17.3, and 183.7 ± 15.0 mm/min, respectively). The two control lines also showed a slightly increased walking velocity at 30°C. As only activation of P1^e^ neurons, but not P1^a^–P1^d^, suppressed feeding, this suppression could not be due to increased locomotion. To further test if feeding and walking velocity were negatively correlated, we plotted all the feeding and locomotor data for all P1-activated males and control males at 25.5°C, 27°C, 28.5°C, and 30°C, and found that feeding and walking velocity were not negatively correlated, and even slightly positively correlated (*r* = 0.37), although the correlation was not significant (*P* = 0.0503). These results indicate that decreased feeding by P1^e^ activation is not due to increased locomotion. How P1^e^ neurons regulate feeding is still unclear and awaits further investigation.

**Fig. 4:**
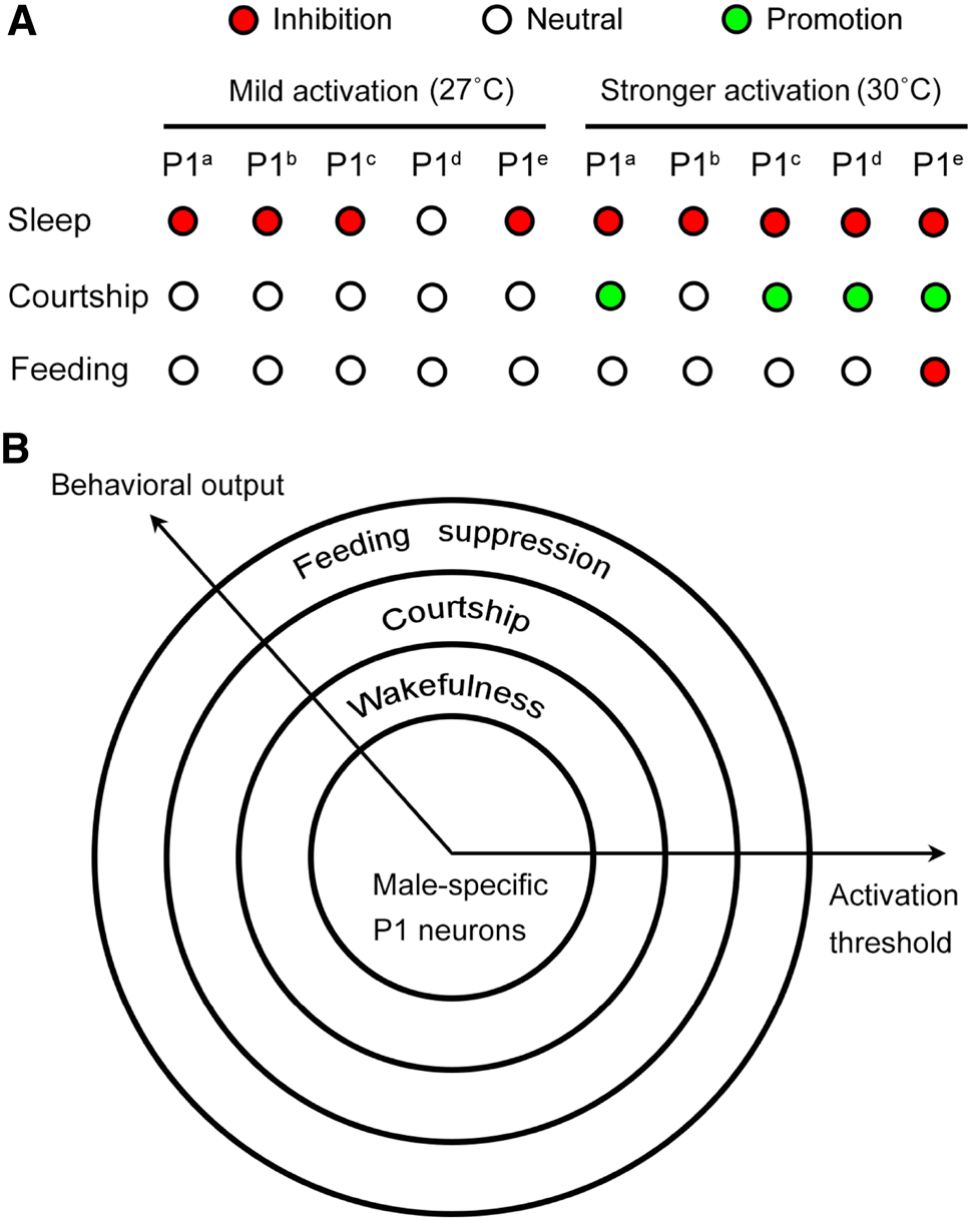
Hierarchical control of sleep, courtship, and feeding by P1 neurons. **A** Summary of the effects of mild and strong activation of P1 neurons using five independent drivers (P1^a^–P1^e^) on sleep, courtship, and feeding behaviors in male flies. **B** Proposed hierarchical model in which different activation thresholds (*e.g*., activation levels, number of neurons) are required for P1 neurons to modulate sleep/wakefulness, courtship, and feeding behaviors.

In summary, we used five independent P1 drivers that potentially labeled partially overlapping and distinct P1 neurons, and systematically investigated how different activation levels (25.5°C, 27°C, 28.5°C, and 30°C) of these targeted P1 neurons affect sleep, courtship, and feeding behaviors. We found that all these behaviors were affected by stronger activation of at least one P1 driver (*e.g.*, P1^e^), suggesting that P1 activity affects all sleep, courtship, and feeding behaviors. Furthermore, we found that differential activation thresholds for P1 neurons were required to affect these three behaviors (Fig. 4A). First, minimum activation (mild activation of 9–12 P1 neurons) was sufficient to suppress sleep; second, stronger activation of P1 neurons was required to induce courtship behavior; and third, only stronger activation of P1^e^ that included ~23 P1 neurons affected feeding.

Sleep, courtship, and feeding are competing behaviors that are mediated by external sensory cues and internal states. Whether these competing behaviors are regulated by common neural nodes is an intriguing question. P1 neurons have been established as a center that mediates sexual arousal, but their role in regulating other internal states and behaviors has been underestimated. Our findings that P1 neurons mediate sleep, courtship, and feeding behaviors not only reveal a neural node (P1) that participates in all these competing behaviors, but also how P1 neurons modulate these behaviors in a hierarchical manner (Fig. 4B).

There are nearly 50 pairs of P1 neurons in the male fly brain [27], and we studied here only 20%–50% of them. Given that mild activation of ~10 P1 neurons was sufficient to inhibit sleep, and stronger activation of ~23 (nearly half) suppressed feeding, what if the other half or all P1 neurons were activated? Do P1 neurons regulate behaviors other than sleep, courtship, aggression, and feeding? Is P1 a center for internal states that coordinate different behaviors? To answer these questions, better tools are needed to subdivide P1 populations, with driver lines that target small and distinct subsets of P1 neurons and driver lines targeting all or the majority of P1 neurons.

We also note that, although males and females play distinct roles in sexual behavior, their differences in non-sexual behaviors (*e.g.*, different amounts of sleep or feeding) are relatively smaller and underestimated, and the mechanism underlying these differences is unclear. That P1 neurons are male-specific and regulate sleep, courtship, aggression, and feeding suggests that sexual dimorphism in these behaviors may be greater than we thought, and our results provide a simple model of how a small number of sex-specific neurons can contribute to various sexually-dimorphic behaviors.

## Electronic supplementary material

Below is the link to the electronic supplementary material.

Supplementary material 1 (PDF 230 kb)
