# Supplementary material for: Hierarchical Control of Drosophila Sleep, Courtship, and Feeding Behaviors by Male-Specific P1 Neurons
Source: Neurosci Bull. 2018 Sep 4;34(6):1105–10. doi: 10.1007/s12264-018-0281-z (PMC6246841; doi:10.1007/s12264-018-0281-z)
Supplement: Supplementary file 1 — Supplementary material 1 (PDF 230 kb) [file 12264_2018_281_MOESM1_ESM.pdf]

Supplementary materials

Hierarchical Control of *Drosophila* Sleep, Courtship, and Feeding Behaviors by Male-specific P1 Neurons

Wenxuan Zhang[1], Chao Guo[1], Dandan Chen[1], Qionglin Peng[1], Yufeng Pan[1,2]

[1]The Key Laboratory of Developmental Genes and Human Disease, Institute of Life Sciences, Southeast University, Nanjing 210096, China

[2]Co-innovation Center of Neuroregeneration, Nantong University, Nantong 226019, China

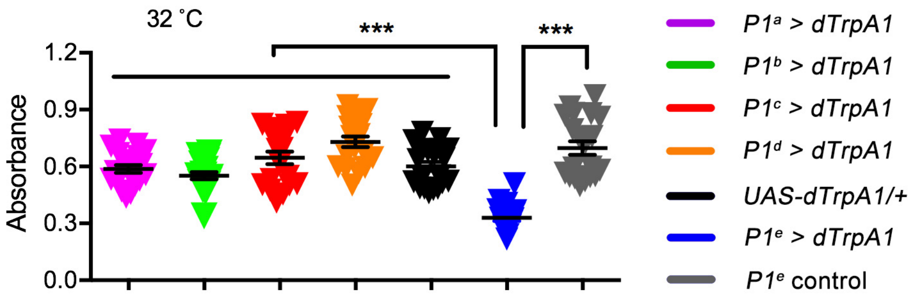

Fig. S1 Feeding behaviors by P1 activated males at 32˚C. Activation of $P1^e$, but not $P1^a$~$P1^d$, significantly suppressed feeding in starved males. n = 20, 20, 19, 20, 20, 18 and 20 respectively (10 flies for each replicate). ***$P < 0.001$, one-way ANOVA. Error bars indicate SEM.
